# Exposure of Hydrophobic Surfaces Initiates Aggregation of Diverse ALS-Causing Superoxide Dismutase-1 Mutants

**DOI:** 10.1016/j.jmb.2010.04.019

**Published:** 2010-06-11

**Authors:** Christian Münch, Anne Bertolotti

**Affiliations:** MRC Laboratory of Molecular Biology, Hills Road, Cambridge CB2 0QH, UK

**Keywords:** superoxide dismutase, amyotrophic lateral sclerosis, aggregation, neurodegeneration

## Abstract

The copper-zinc superoxide dismutase-1 (SOD1) is a highly structured protein and, *a priori*, one of the least likely proteins to be involved in a misfolding disease. However, more than 140, mostly missense, mutations in the *SOD1* gene cause aggregation of the affected protein in familial forms of amyotrophic lateral sclerosis (ALS). The remarkable diversity of the effects of these mutations on SOD1 properties has suggested that they promote aggregation by a variety of mechanisms. Experimental assessment of surface hydrophobicity using a sensitive fluorescent-based assay, revealed that diverse ALS-causing mutations provoke SOD1 aggregation by increasing their propensity to expose hydrophobic surfaces. These findings could not be anticipated from analysis of the amino acid sequence. Our results uncover the biochemical nature of the misfolded aggregation-prone intermediate and reconcile the seemingly diverse effects of ALS-causing mutations into a unifying mechanism. Furthermore, the method we describe here will be useful for investigating and interfering with aggregation of various proteins and thereby provide insight into the molecular mechanisms underlying many neurodegenerative diseases.

## Introduction

Aggregation of proteins of unrelated sequence is a major hallmark of neurodegenerative diseases. Amyotrophic lateral sclerosis (ALS) is the most common motor neuron disease. It has an adult onset and is rapidly progressive. The majority of ALS cases are sporadic but a large group of dominantly inherited forms of the disease are caused by mutations in the gene encoding the abundantly expressed cytosolic superoxide dismutase-1 (SOD1).^1,2^ How SOD1 mutants provoke motor neuron degeneration remains to be elucidated, but it is now well established that SOD1 mutations cause familial ALS (fALS) by a gain of toxic properties and not by a loss of enzymatic activity. Mice lacking SOD1 do not develop motor neuron disease.^3^ In contrast, transgenic mice expressing the ALS-causing SOD1 mutants, in addition to their endogenous SOD1, develop symptoms reminiscent of the human disease.^1,4^ Similar to other neurodegenerative diseases, a major hallmark of ALS is the presence of proteinaceous inclusions in affected neurons. In fALS patients with SOD1 mutations, aggregated SOD1 is the major component of the motor neuron inclusions^5,6^ and ALS-causing SOD1 mutants also form inclusions in transgenic mice.^1,7^ Thus, ALS-causing mutations confer aggregation propensity as well as some toxic properties to SOD1. Understanding how SOD1 mutations cause aggregation of the protein is of major importance, as it is one of the earliest events in the ALS pathogenesis.

SOD1 is an abundant and ubiquitously expressed 32 kDa homodimeric enzyme that catalyzes the dismutation of superoxide radicals. Each monomer folds as an eight-stranded Greek key β-barrel,^2^ binds one copper and one zinc ion, and contains a disulfide bond. The native protein is extremely stable and thus, *a priori,* one of the least likely proteins to be involved in a neurodegenerative disease. However, more than 140, mostly missense mutations in *SOD1* cause fALS.^2^ SOD1 mutations are scattered through the entire sequence of the protein and have very diverse effects on the properties of the protein. SOD1 mutants partition in two groups, on the basis of their metal-binding properties and biological activities. One group is characterized by mutations in the metal-binding region (MBR) that perturb metal binding. The other fALS mutants generally retain wild-type metal content and biological activity and are referred to as wild-type like mutants (WTL).^2^ How such diverse ALS-causing SOD1 mutations all cause aggregation of the protein is a great puzzle. Most mutations in SOD1 only marginally reduce the stability of the native protein^8^ and a variety of different mechanisms have been proposed to explain the increased aggregation propensity of the diverse SOD1 mutants. Aggregation can arise as a consequence of perturbation of the structural integrity due to loss of metal binding,^8–12^ reduction of the intra-monomer disulfide bond^13–16^, oxidation,^17^ destabilization of the dimer^18,19^ or alteration of post-translational modifications.^20,21^ Some biochemical properties of the mutant proteins such as reduction of the repulsive charge of the protein,^22^ aberrant hydrophobicity of the apo-protein,^23^ perturbed folding^24,25^ or increased unfolding rates^24,26^ could account for the increased aggregation of SOD1 mutants, but it has not been established whether any of these alterations directly cause aggregation. Because of the diversity of the alterations provoked by SOD1 mutations, specific rules have been proposed to govern aggregation of distinct subsets of ALS-causing mutants.

Some structural alteration of the native fold ought to be required to elicit SOD1 misfolding. However, because SOD1 is such a stable protein, aggregation is likely to be initiated by local and possibly subtle unfolding of the native state, rather than global unfolding. Therefore, our ability to detect early conformational changes on the misfolding pathway depends on the availability of a sensitive assay. While using the conformation-sensitive dye Sypro Orange, which fluoresces in a hydrophobic environment,^27^ as a sensitive method to detect conformational transition, we have uncovered the biochemical nature of the aggregation-prone conformer. We found that ALS-causing mutations provoke aggregation by increasing the propensity of diverse SOD1 mutants to expose hydrophobic surfaces, a common feature that could not be anticipated from bioinformatic analysis of the biochemical alterations provoked by the mutations.^28^

## Results

### Increased Sypro Orange fluorescence is a common feature of ALS-causing SOD1 mutants

**Fig. 1:**
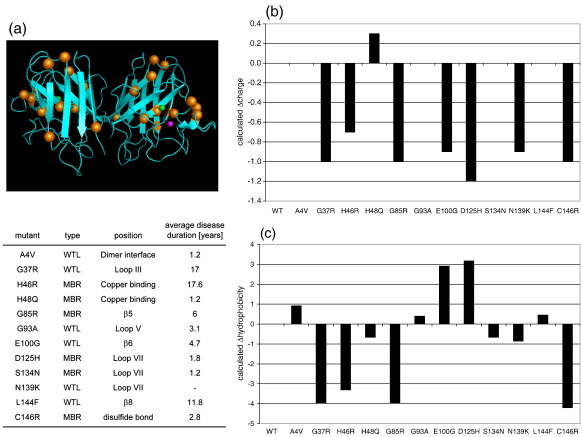
Diverse properties of fALS-causing SOD1 mutants. (a) Schematic representation of SOD1 structure (PDB entry 1SPD).^53^ Mutants analyzed in this study are highlighted in orange, copper in green and zinc in magenta. Table listing key features of the mutants analyzed in this study. (b) Calculated change of charge (Δcharge) resulting from the mutations. Calculations were performed with PROTEIN CALCULATOR v3.3. (c) Calculated change of hydrophobicity (Δhydrophobicity) resulting from the mutations. Hydrophobicity values were calculated according to,^42^ with hydrophobicity values taken form.^54^

We selected a set of structurally diverse SOD1 mutants, including mutants that account for most of the fALS cases with SOD1 mutations.^29^ The selected mutants display the broad variety of biophysical and biochemical properties that characterize fALS-causing SOD1 mutations and different clinical characteristics (Fig. 1a). This comprises six WTL and six MBR mutants selected because 1) they all cause fALS, 2) their positions are scattered throughout the sequence of the protein, 3) they have been previously characterized and exhibit different enzymatic, physicochemical and structural properties, 4) the disease duration varies greatly for patients with these different SOD1 mutations (Fig. 1a). The selected mutants include the A4V mutant, which is both the most common and the most severe fALS-causing SOD1 mutant and retains WTL properties;^30^ the common H46R mutant with compromised copper binding and disease duration of about 17 years;^31^ and the N139K and S134N mutants, which were previously described as nearly indistinguishable from the wild-type protein.^2,10^ A bioinformatic analysis has previously revealed that SOD1 mutations have an overall tendency to decrease the net negative charge of SOD1.^22^ We also find this trend in the representative set of SOD1 mutants selected in this study, since seven out of the twelve mutants selected exhibited a decrease in their repulsive charge (Fig. 1b). In addition, we examined the change of hydrophobicity introduced by the mutations. Four mutations dramatically decreased and two increased the hydrophobicity, while the other mutations only caused subtle changes (Fig. 1c). These analyses confirmed that the selected SOD1 mutants cover the broad range of properties characteristic of SOD1 mutants.

**Fig. 2:**
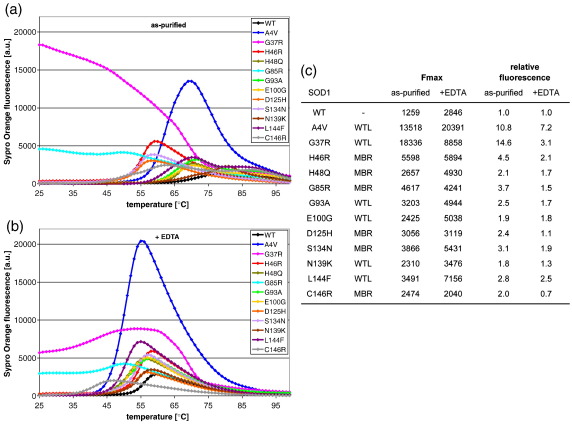
Increased surface hydrophobicity upon thermal denaturation is a generic feature of ALS-causing SOD1 mutants. (a) Sypro Orange fluorescence during thermal unfolding of as-purified SOD1 without or (b) with 20 mM EDTA. Data are means of 3 independent experiments. s.d. are presented in Supplementary Fig. 2. (c) Hydrophobicity values measured in (a and b), as the mean Sypro Orange-derived fluorescence maxima (Fmax) or normalized to the Fmax of SOD1^WT^, using either as-purified or EDTA-treated proteins.

Aiming to set up an assay to detect the conformational rearrangements on the misfolding pathway, we analyzed the behaviour of SOD1 mutants in the presence of a conformation sensitive dye. Proteins were expressed in insect cells and purified as described^9^ (and Supplementary Fig. 1). We carried out thermal denaturation of SOD1 proteins in the presence of Sypro Orange (Fig. 2a, b and Supplementary Fig. 2). At room temperature, SOD1^G37R^ and SOD1^G85R^ already exhibited a significant fluorescence in the presence of the conformation sensitive dye, in contrast to all other mutants and wild-type protein (Fig. 2a). This confirmed that under native conditions, SOD1^WT^ and most mutants did not expose hydrophobic surfaces as expected for tightly folded proteins. Heat denaturation of all mutants other than SOD1^G37R^ and SOD1^G85R^, in the presence of Sypro Orange, resulted in a marked increase in fluorescence. Notably, the assay used here revealed differences between SOD1^WT^ and the N139K and S134N mutants that have escaped previous analyses. The fluorescence maxima of the as-purified SOD1 mutants ranged from about 2-fold, to more than a 14-fold increase relative to the wild-type protein (Fig. 2a and c). We defined relative surface hydrophobicity values as the increase in fluorescence maxima relative to wild-type SOD1 (Fig. 2c). This revealed that the structurally diverse SOD1 mutants exposed more hydrophobicity than the wild-type protein.

SOD1 variants expressed and purified in similar conditions as used here were found to exhibit various metal contents^9^ and previous studies have revealed that metal deficiency increases hydrophobicity.^23,32^ We next set up to determine the metallation status of most of the SOD1 variants used in this study, in order to determine whether the differences of hydrophobicity of the diverse SOD1 variants reflected differences in metal content. In agreement with a previous study, all the purified SOD1 variants analyzed had substochiometric copper content and the MBR mutants H46R, S134N and D125H were both copper- and zinc-deficient (Supplementary Fig. 3a and^9^). In the other mutants analyzed, the zinc site was fully occupied (Supplementary Fig. 3a). We failed to detect a correlation between low metal content and high amount of exposed hydrophobic surfaces (Supplementary Fig. 3b). However, it has long been suspected that metal deficiency could be at the origin of the pathogenicity of SOD1 mutants.^8,9^ We next exposed the purified SOD1 variants to 20 mM EDTA and monitored the effects of such treatment. As expected, the thermal denaturation profiles, in the presence of Sypro Orange, of the metal deficient mutants H46R, S134N and D125H, were very similar with or without 20 mM EDTA (Fig. 2a-c). The same was observed for the MBR mutant C146R. In contrast, the fluorescence maxima of the other mutants were higher when heat denatured in the presence of EDTA (Fig. 2a-c). Thus, EDTA treatment significantly reduced the metal content of the SOD1 variants and this reduction further enhanced exposure of hydrophobic surfaces of destabilized SOD1 mutants. As observed for the as-purified proteins, most EDTA-treated SOD1 mutants also exhibited fluorescence maxima that were higher than that of the wild-type protein (Fig. 2b and c). However, EDTA treatment attenuated the differences in surface hydrophobicity between the wild-type protein and the mutants (Fig. 2a-c). This is likely due to the finding that demetallation increased exposure of hydrophobic surfaces on SOD1^WT^ (Fig. 2a-c).

Environmentally sensitive fluorescent dyes have been used previously to monitor thermal unfolding.^34^ The transition midpoint of the denaturation curves indicates the unfolding temperature (Tm). The apparent Tm values of the EDTA treated proteins were extracted from the Sypro Orange analyses (Supplementary Fig. 3c). We found that the apparent Tm values of our EDTA-treated mutants were nearly identical to the ones published for the same mutants in the apo-state (Supplementary Fig. 3c and^10,33^). This reveals that our EDTA-treated proteins are similar to apo-proteins prepared in other studies. As expected, EDTA treatment dramatically reduced the Tm of the biologically metallated proteins but had a minor effect on the metal deficient proteins H46R, S134N and D125H. Interestingly, except for SOD1^N139K^, all as-purified SOD1 mutants had a lower apparent Tm than the wild-type protein (Supplementary Fig. 3c). We then analyzed whether the extent of exposure of hydrophobicity directly correlated with Tm. While thermal unfolding of most SOD1 mutants is required to expose hydrophobic surfaces, the amount of exposed hydrophobic surfaces did not correlate directly with the apparent Tm of the different mutants (Supplementary Fig. 3d). This revealed that the knowledge of Tm can't predict the amount of surface hydrophobicity exposed by each mutant.

Together, these results reveal that exposure of hydrophobic surfaces is a common feature of diverse SOD1 mutants with different metal content and stability, when subjected to denaturation.

### Increased surface hydrophobicity correlates with aggregation of SOD1^A4V^

**Fig. 3:**
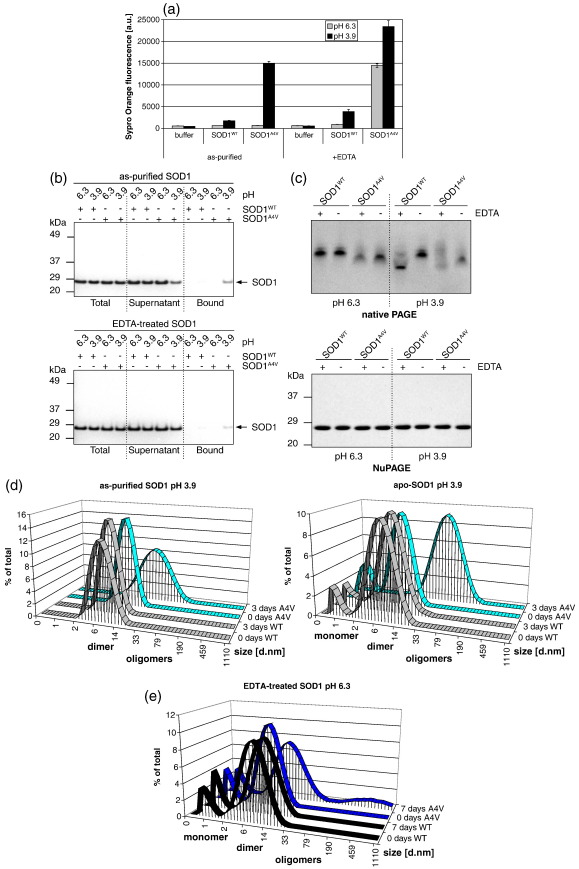
Destabilization of as-purified SOD1^A4V^ by EDTA or low pH exposes hydrophobic surfaces and triggers aggregation. (a) Assessment of surface hydrophobicity by Sypro Orange fluorescence. Means and s.d. of replicate experiments (n = 3) are shown. (b) NuPAGE analysis of SOD1 binding to hydrophobic beads. (c) Electrophoretic mobilities of the SOD1 variants on native PAGE (1.2 μg) or denaturing NuPAGE (0.75 μg). Proteins were treated as indicated 30 min prior electrophoresis. (d, e) Aggregation of SOD1^A4V^ monitored by DLS. d.nm: Diameter in nm.

Having found an unprecedented common feature for these diverse SOD1 mutants we then asked whether increased exposure of hydrophobic surfaces caused aggregation. We used conditions previously established to elicit mutant SOD1 aggregation to address this question, such as acidic pH in the presence or absence of a chelator. In buffer alone, or in the presence of as-purified SOD1^WT^ or SOD1^A4V^, Sypro Orange fluorescence was very low, indicating that hydrophobic residues are buried in both SOD1^WT^ and SOD1^A4V^, as expected for native proteins (Fig. 3a). At physiological temperature, Sypro Orange fluorescence increased dramatically only in the presence of EDTA-treated SOD1^A4V^, indicating that EDTA-treated SOD1^A4V^ exhibited greater surface hydrophobicity than the SOD1^WT^ exposed to the same condition, or the untreated proteins (Fig. 3a), as previously observed.^13^

Proteins were next exposed to low pH, a condition that elicits SOD1^A4V^ aggregation, with or without EDTA.^35^ At pH 3.9, both EDTA-treated and as-purified SOD1^A4V^ became strongly fluorescent in the presence of Sypro Orange, unlike SOD1^WT^ exposed to the same conditions (Fig. 3a), revealing that EDTA or low pH exposed hydrophobic surfaces on SOD1^A4V^ but not on the wild-type protein. To confirm these findings, SOD1^WT^ and SOD1^A4V^ were next incubated on Phenyl Sepharose. After extensive washing, a fraction of EDTA-treated and as-purified SOD1^A4V^ exposed to low pH was selectively retained on the hydrophobic resin (Fig. 3b). These results confirm that the fluorescence-based assay is a highly sensitive method for detecting surface hydrophobicity.

To assess whether exposure to acidic pH in the presence of EDTA efficiently demetallated the proteins, SOD1^WT^ and SOD1^A4V^ were analyzed on native PAGE, since this method was shown to resolve differentially metallated species.^9^ The mobility of as-purified SOD1^A4V^ was faster than as-purified SOD1^WT^ (Fig. 3c), in good agreement with the finding that this mutant had reduced copper content, compared to the wild-type protein (Supplementary Fig. 3a). At pH 6.3, EDTA-treated SOD1^A4V^ exhibited the same mobility as the untreated mutant on native PAGE (Fig. 3c), while Sypro Orange fluorescence increased in the presence of EDTA-treated SOD1^A4V^, but not the untreated mutant (Fig. 3a). This reveals that Sypro Orange fluorescence detected changes that were not detectable by native PAGE, suggesting that the Sypro Orange detects conformational changes that precede metal loss. At acidic pH, EDTA treatment increased mobility of both SOD1^WT^ and SOD1^A4V^ on native PAGE, indicating that such treatments efficiently depleted metal and produced apo-proteins (Fig. 3c). As previously observed,^36^ we also find that demetallation destabilized SOD1 dimers (Fig. 3d and e).

We next carried out analyses of SOD1 aggregation, using as-purified and apo-proteins at a 10 μM concentration.^33^ Aggregation was monitored by dynamic light scattering (DLS), which revealed that after 3 days at pH 3.9, both apo- and as-purified SOD1^A4V^ were aggregated (Fig. 3d). As previously observed for wild-type SOD1^WT^, we found that low pH destabilized both SOD1^WT^ and SOD1^A4V^ dimers,^37^ but only apo-SOD1^A4V^ aggregated. Under the same conditions, apo-SOD1^A4V^ dramatically increased Sypro Orange fluorescence (Fig. 3a). In good agreement with the high fluorescence of demetallated SOD1^A4V^ at low pH, compared to the as-purified protein (Fig. 3a), we observed that apo-SOD1^A4V^ formed larger particles than as-purified SOD1^A4V^ (Fig. 3d). After 7 days, EDTA-treated SOD1^A4V^ also aggregated at pH 6.3 (Fig. 3e). In contrast, the wild-type protein remained soluble in each condition tested (Fig. 3d, e, Supplementary Fig. 4 and data not shown). All together, these results show that increased exposure of hydrophobic surfaces correlates with aggregation of SOD1^A4V^.

### TFE exposes hydrophobic surfaces and provokes aggregation of SOD1^H46R^

**Fig. 4:**
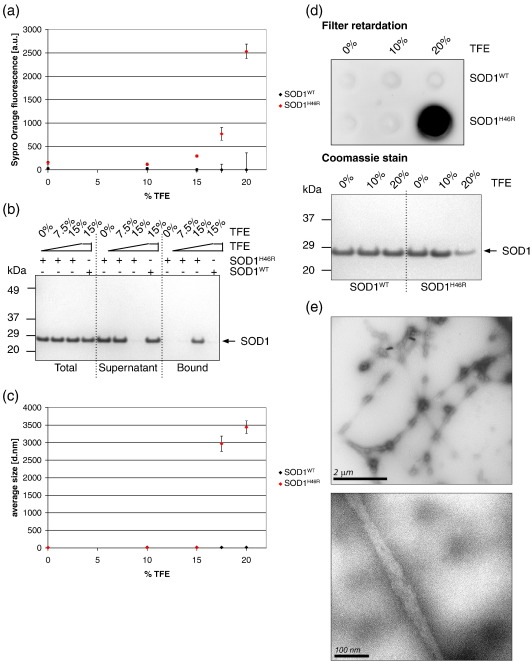
TFE treatment exposes hydrophobic surfaces on as-purified SOD1^H46R^ and provokes fibrillogenesis. (a) Assessment of SOD1^H46R^ surface hydrophobicity by Sypro Orange fluorescence in the presence of increasing TFE concentrations. (b) NuPAGE analysis of SOD1 binding to hydrophobic beads. (c) Aggregation of SOD1^H46R^ monitored by DLS. (d) Aggregation of SOD1^H46R^ revealed by filter retardation assay and NuPAGE analysis of aliquots of the samples used in the filter retardation assay. (e) Electron micrographs of negatively stained SOD1^H46R^ fibrils formed in 20% TFE acquired at magnifications of 7100 (upper panel) and 52000 (lower panel). Means and s.d. of 3 independent experiments are shown in (a and c).

We next examined whether similar properties underlie aggregation of a mutant unrelated to the WTL mutant SOD1^A4V^. As-purified SOD1^WT^ and the MBR mutant SOD1^H46R^ were exposed to increasing trifluoroethanol (TFE) concentrations, a condition previously shown to trigger aggregation of SOD1 mutants,^33^ as well as other globular proteins.^38^ TFE increased Sypro Orange fluorescence (data not shown). However, when the fluorescence of the dye in buffer was subtracted, we found that the fluorescence of SOD1^H46R^, but not SOD1^WT^, increased dramatically in Sypro Orange, with increasing TFE concentrations (Fig. 4a). This result indicates that in the presence of TFE, SOD1^H46R^ but not SOD1^WT^ exposed hydrophobic surfaces, as confirmed by the specific retention of SOD1^H46R^, in the presence of TFE, on hydrophobic resin (Fig. 4b). In parallel, aggregation was monitored by DLS and revealed that TFE concentrations higher than 15% triggered aggregation of SOD1^H46R^, but not SOD1^WT^ (Fig. 4c).

To confirm that the large particles observed upon incubation of SOD1^H46R^ with TFE were aggregates, SOD1^WT^ and SOD1^H46R^, in non-denaturing buffer or in the presence of increasing concentrations of TFE, were filtered through a cellulose acetate membrane and revealed with SOD1 antibodies, as described.^39,40^ While unaggregated SOD1 filtered through the membrane, aggregated SOD1^H46R^ was retained when the mutant was exposed to 20% TFE (Fig. 4d), indicating that the large particles observed by DSL were SOD1^H46R^ aggregates (Fig. 4c). Equal amounts of proteins were loaded on the filter retardation assay and on a denaturing NuPAGE gel (Fig. 4d, lower panel). However, the amount of SOD1^H46R^ in 20% TFE that was resolved on the denaturing gel was consistently lower than that in absence of TFE. This indicated that aggregates were not resolved on the gel, suggesting that they might be resistant to boiling in reducing buffer. We noticed that SOD1^H46R^ required lower TFE concentrations to trigger Sypro Orange fluorescence than to aggregate (Fig. 4a, c and d). This indicates that exposure of hydrophobic surfaces precedes aggregation.

We next examined the morphology of mutant SOD1 aggregates to determine whether they were amorphous or ordered. Electron microscopy revealed that SOD1^H46R^ aggregates exhibited both fibrillar and granular components, reminiscent of the granule-coated fibrils found in affected neurons of ALS patients (Fig. 4e and Ref. 41). Together, these results demonstrate that aggregation of two distinct SOD1 mutants, elicited under different conditions, directly correlates with exposure of hydrophobic surfaces.

### Exposure of hydrophobic surfaces precedes aggregation

**Fig. 5:**
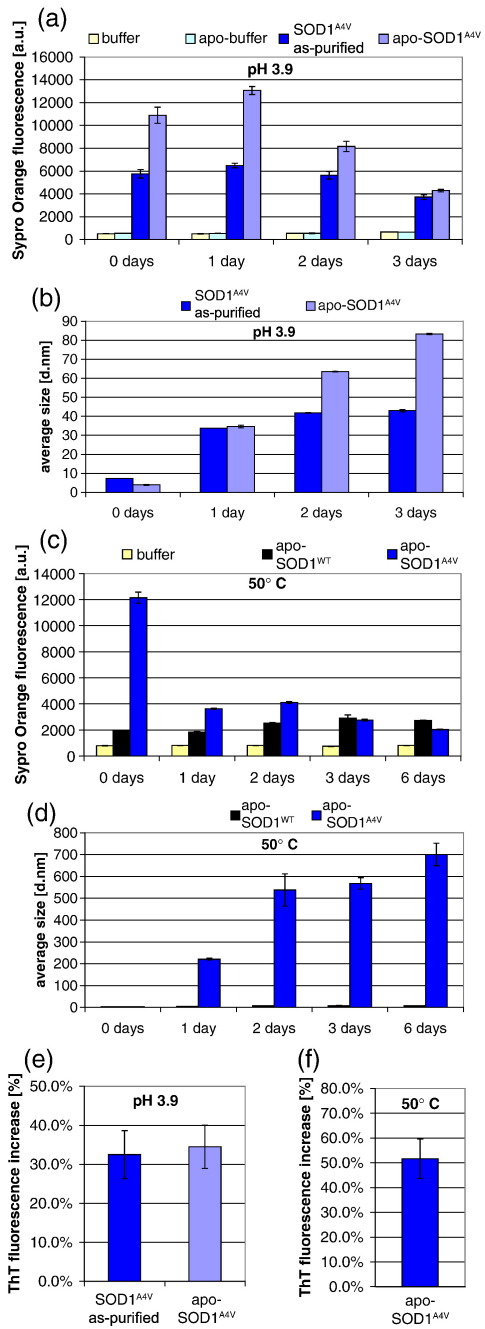
Aggregation of as-purified SOD1 mutants follows exposure of hydrophobic surfaces. (a) Time course of measurements of exposed hydrophobicity monitored by Sypro Orange fluorescence and (b) aggregation of SOD1^A4V^ at acidic pH monitored by DLS. Note that demetallation first provoked a decrease in the size of particles measured, indicating that metal loss provoked monomerization of the protein. (c) Time course of measurements of exposed hydrophobicity and (d) aggregation of apo-SOD1^A4V^ exposed to 50 °C. (e, f) aggregation of SOD1^A4V^ monitored by ThT after 3 days of incubation at acidic pH (e) or 50 °C (f). Data are means and s.d. values of replicate experiments (n = 3).

Aggregation of SOD1^H46R^ occurred within minutes after TFE addition, while a longer time was required to detect aggregation of SOD1^A4V^ at low pH (Figs. 3, 4 and data not shown). We took advantage of the slow aggregation kinetics of both as-purified and apo-SOD1^A4V^ at acidic pH to analyze the aggregation process in greater detail. As-purified and apo-SOD1^A4V^ were adjusted to low pH (day 0) and aliquots taken every day to analyze both Sypro Orange-derived fluorescence and aggregation. Immediately after exposure to low pH, both as-purified and apo-SOD1^A4V^ increased the fluorescence of the conformation-sensitive dye (Fig. 5a, day 0). The increase in fluorescence preceded the onset of aggregation (Fig. 5a and b, day 0). Fluorescence of Sypro Orange then reached a maximum after 1 day and gradually decreased after 2 days (Fig. 5a). Concomitantly, aggregates gradually grew (Fig. 5b and Supplementary Fig. 5a). Note that the soluble proteins were barely detectable after 2 days (Supplementary Fig. 5a). Together, these analyses reveal that low pH exposes hydrophobic regions on the surface of SOD1^A4V^ and such regions are buried in aggregates.

To determine whether these findings were restricted to the experimental conditions used to induce mutant SOD1 aggregation or were inherent to the aggregation mechanism, we used high temperature, since this condition was previously found to trigger SOD1^A4V^ aggregation.^33^ Similar to what we observed upon destabilization of SOD1^A4V^ at low pH, heating SOD1^A4V^ to 50 °C increased the fluorescence of Sypro Orange (Fig. 5c). The fluorescence of the conformation-sensitive dye decreased over time, while aggregates grew (Fig. 5c and d). Note that no soluble proteins were detectable after 1 day (Supplementary Fig. 5c). In contrast, heat did not provoke fluorescence of SOD1^WT^ or aggregation (Fig. 5c and d). Like the ALS deposits, 3 days-old aggregates were amyloid-like since they increased the fluorescence of Thioflavin T (ThT), an amyloid specific probe (Fig. 5e and f). In contrast, SOD1^WT^ didn't increase ThT fluorescence (data not shown). The ThT increase for SOD1 aggregates was modest, in agreement with previous studies.^33,35^ Congo Red binding experiments also suggested that SOD1^A4V^ aggregates, in contrast to the wild-type protein, contain an amyloid component, since they produced a small but reproducible shift in the absorbance spectra of Congo Red (Supplementary Fig. 5b, d), as previously reported.^33,35^ Taken together, these results indicate that denaturation of SOD1 mutants by diverse treatments exposes hydrophobic surfaces and that such regions engage non-native interactions to form amyloid-like aggregates. Thus, ordered assembly of SOD1 aggregates is likely to be driven by hydrophobic interactions. These experiments also reveal that Sypro Orange-derived fluorescence is a marker of one of the earliest conformational rearrangement in the aggregation pathway, rather than amyloids per se.

### Increased propensity of diverse ALS-causing SOD1 mutants to expose hydrophobic surfaces causes aggregation

**Fig. 6:**
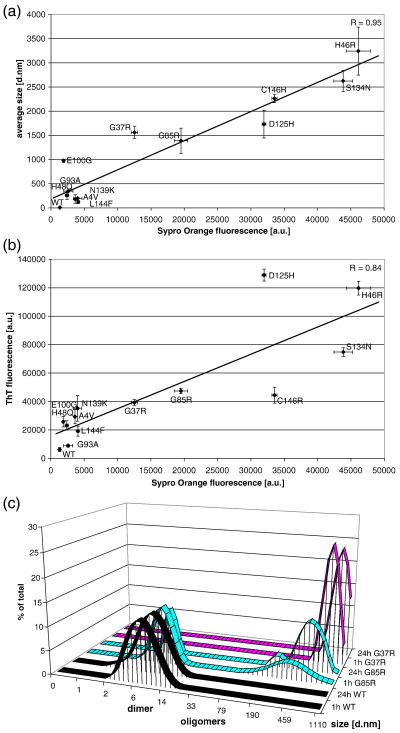
Aggregation of as-purified ALS-causing SOD1 mutants is caused by their increased propensity to expose hydrophobic surfaces. (a, b) Correlation between surface hydrophobicity measured by Sypro Orange fluorescence and aggregation measured by DLS (a) and ThT fluorescence (b). Data are means and s.e.m. from 3 independent experiments. Measurements were made 20 minutes after TFE treatment. The high linear correlation coefficient R, denotes the strength of the correlation. Note that the MBR mutants are more susceptible to TFE-induced aggregation than the WTL mutants. (c) Aggregation of SOD1^G85R^ and SOD1^G37R^ at room temperature, revealed by DLS.

In all conditions examined using two specific SOD1 mutants, we observed a correlation between exposure of hydrophobic surfaces and aggregation. To determine whether increased exposure of hydrophobic surfaces correlates with aggregation of diverse SOD1 mutants, we monitored aggregation and exposure of hydrophobic surfaces of the representative set of 12 mutants. As-purified SOD1^WT^ and mutant proteins were exposed to 20% TFE, a condition that was found to elicit aggregation of the diverse SOD1 mutants, but not the wild-type protein. This treatment converted the soluble MBR mutants into aggregates in 20 minutes and was also efficient to provoke aggregation of the WTL mutants (Supplementary Fig. 6a). Exposure of surface hydrophobicity was monitored by Sypro Orange fluorescence and aggregation monitored both by DLS and ThT fluorescence (Fig. 6a and b). ThT fluorescence indicated that aggregates have an amyloid component. Analysis of Congo Red binding confirmed this (Supplementary Fig. 6b). We found exposure of hydrophobicity and ordered assembly of SOD1 aggregates, monitored by these two independent methods, were strongly correlated. The strength of the correlation between exposure of hydrophobic surfaces and aggregation is attested by the very high linear correlation coefficient (Fig. 6a, R = 0.95 and b, R = 0.84). This revealed that exposure of hydrophobicity is a common feature that provokes aggregation of diverse SOD1 mutants.

To further demonstrate that the exposure of hydrophobic surfaces predicts aggregation, we focused on SOD1^G37R^ and SOD1^G85R^. These two mutants produced high Sypro Orange fluorescence at room temperature (Fig. 2a), suggesting that they might aggregate at room temperature. DLS analyses confirmed this hypothesis (Fig. 6c).

## Discussion

A common feature of diverse neurodegenerative diseases is the deposition of aggregated proteins in affected neurons. Mutations of natively unstructured proteins associated with familial forms of these diseases cause aggregation of the proteins by altering simple intrinsic physicochemical properties such as hydrophobicity, secondary structure and charge.^42,43^ However, the rules governing aggregation propensity of highly structured proteins, such as SOD1, remained unknown. The results presented here reveal that increased surface hydrophobicity is a generic feature of structurally diverse ALS-causing SOD1 mutants that could not be anticipated by calculating the changes in hydrophobicity caused by the mutation or by using algorithms designed to predict protein aggregation.^28^ This common property is intrinsic to fALS mutants but not necessarily constitutive. The destabilizing mutations G85R and G37R constitutively expose hydrophobic surfaces. For the other SOD1 mutations, destabilization of the proteins exposes otherwise buried hydrophobic surfaces and causes aggregation. Previous studies have shown that SOD1 mutations increase unfolding rates^24,26,33^ and we found here that such unfolded intermediates expose aggregation-prone surfaces. While it is expected that partly unfolded proteins expose some hydrophobic surfaces, and such hydrophobic regions exist in SOD1,^44^ it could not be anticipated that diverse ALS-causing SOD1 point mutations, causing a variety of alterations on the properties of the proteins, all increase the propensity to expose hydrophobic surfaces and that this common feature is the molecular determinant underlying the formation of the ordered aggregates. Furthermore, the effects of the mutations analyzed here on the predicted hydrophobicity of the protein are completely random: Some mutations increase and some decrease hydrophobicity.

It has been previously reported that decreased stability and metal deficiency are important factors that influence SOD1 aggregation.^8,9^ We did not observe a direct correlation between metal content and exposure of hydrophobicity upon thermal denaturation of the as-purified proteins (Supplementary Fig. 3). However, treatment with a chelator further increased exposure of hydrophobic surfaces of the SOD1 variants that where loaded with metal. This indicates that demetallation increases exposure of hydrophobicity and thereby increases aggregation propensity. The MBR mutants were also more susceptible to TFE-induced aggregation than the WTL (Fig. 6), suggesting that these mutants are more vulnerable to destabilization. Similar to what we observed with metal contents, we found no direct correlation between Tm and exposed hydrophobicity (Supplementary Fig. 3). Yet, destabilization of the natively stable mutants is required to expose the otherwise buried hydrophobic surfaces. These observations are consistent with the following interpretation: metal deficiency, stability, susceptibility to unfolding are likely to be interconnected. Destabilization of metallated proteins is likely to provoke metal loss; conversely metal deficiency destabilizes the proteins. We found here that destabilizing diverse SOD1 mutants by various treatments systematically increased exposure of hydrophobic surfaces on the mutant but not the wild-type protein.

Taking advantage of the slow aggregation kinetics of SOD1 mutants at low pH and high temperature, we found that exposure of hydrophobic surfaces precedes aggregation (Fig. 5). This shows that aggregation of diverse pathogenic SOD1 mutants is driven by intermolecular hydrophobic interactions either between constitutively hydrophobic mutants or aggregation intermediates exposing hydrophobic surfaces. Our results are consistent with the structures of SOD1 amyloid-like filaments that have revealed that these amyloids are formed after local unfolding of the zinc and electrostatic loops that creates a hydrophobic interface between two proteins.^45^ In two previous studies, a subset of SOD1 mutants from mice tissues have been captured on hydrophobic resins.^23,46^ Our findings reveal that exposure of hydrophobic surfaces is a generic feature of diverse SOD1 mutants and this property governs aggregation propensity. The increased surface hydrophobicity of SOD1 mutants may also account for several of their properties: Their perturbed folding, both in vitro and in cells,^24,25^ their selective association with the cytoplasmic face of mitochondria,^47^ their recognition by chaperones^48^ as well as their decreased half-life,^49^ since hydrophobic surfaces, once recognized by chaperones may target the protein to the degradation machinery.^50^

Whether fALS with SOD1 mutations is a typical amyloidosis is not clear. Mutant SOD1 inclusions in human tissues are composed of 15- 25 nm granules-coated fibrils^41^ but they are not revealed by amyloid-specific dyes. However, inclusions are Thioflavin-S-positive in mice expressing SOD1 mutants.^39^ In vitro, we found that SOD1 aggregates increased ThT fluorescence, shifted Congo Red absorbance towards 541 nm and that they exhibited both fibrillar and granular components (Fig. 4e). Previous studies have also reported that SOD1 aggregates contain an amyloid component.^11,15,33,35^ Since minute amounts of misfolded SOD1 cause ALS,^51^ one hypothesis that can reconcile these seemingly conflicting observations is as follows: SOD1 aggregates contain an amyloid component but its low abundance precludes its detection by amyloid dyes in human tissues.

Aggregation-prone hydrophobic surfaces are only transiently exposed on the aggregation intermediate and later buried inside the aggregate. These findings may provide a molecular basis for the hypothesis of the toxic aggregation intermediate. Exposure of hydrophobic surfaces on the aggregation intermediate may be detrimental to the cell, while the mature fibrillar aggregate, burying these surfaces, is predicted to be less harmful. In aggregation reactions with slow kinetics, we found that Sypro Orange fluorescence precedes aggregation and ThT fluorescence (Fig. 5) indicating that Sypro Orange is a marker of an early species in the aggregation pathway rather than another amyloid marker. Sypro Orange reveals a soluble, yet aggregation-prone conformer that builds up aggregates. When aggregation is elicited with TFE, Sypro Orange remains high in the presence of TFE-treated SOD1 mutants after the onset of aggregation (data not shown). Since aggregation is observed rapidly after TFE addition, it is likely that TFE is a more potent destabilizing agent than pH or temperature. This suggests that under harsh conditions, some hydrophobic surfaces remained exposed on the aggregates, in contrast to the aggregation elicited in milder conditions such as low pH or high temperature (Fig. 5). Whether the aggregation pathways are different under different aggregation conditions is unclear. Regardless of the aggregation pathway, we found here that destabilization of SOD1 mutants, by several methods, exposes hydrophobic surfaces on the mutant proteins but not the wild-type. This reveals that, the initiation of aggregation is conserved. We propose that, any condition, yet to be identified, that will destabilize the protein in motor neurons, will expose hydrophobic surfaces and trigger the ordered assembly of fALS-causing SOD1 mutants.

This study identifies the common nature of the early aggregation-prone conformer of diverse ALS-causing SOD1 mutants and thereby provides essential information to elucidate the mechanism by which it provokes motor neuron death and to interfere with this process.

The method used here is broadly applicable, quantitative and high-throughput. The fluorescent-based assay can be used to screen for compounds that prevent the formation of the aggregation-prone conformer, one of the earliest events in the disease process. In addition, this method will be useful for investigating the aggregation propensity of a wide variety of proteins and thereby provide insight into the molecular mechanisms underlying many neurodegenerative diseases.

## Materials and methods

### Protein purification

cDNAs encoding human SOD1 mutants were generated by polymerase chain reactions. Wild-type and mutant cDNAs were cloned in pFastBac1 (Invitrogen) and expressed in Sf9 cells in the presence of 150 μM CuCl_2_ and ZnCl_2_. SOD1 proteins were purified as described.^9^ Assays were performed in the commonly used 10 mM MES buffer pH 6.3^8^, with or without 20 mM EDTA to generate apo-SOD1. In Figs. 3 and 5 (a and b), 50 mM MES buffer pH 6.3 or sodium acetate buffer pH 3.9, were used. Protein concentrations were determined by measuring OD at 280 nm and by quantitative amino acid analyses.

### Fluorescence measurements

Equal amounts of protein (3 μg in Figs. 2, 3, 5 and 6; 1 μg in Fig. 4) were mixed with an excess of Sypro Orange (10 × concentration of S5692 Sigma-Aldrich) in 20 μl in a 96 well plate and fluorescence was measured in the 7900HT Fast Real-Time PCR System from Applied Biosystems and expressed as arbitrary units (a.u.). Thermal denaturations were conducted with a heating rate of 1 °C/1.5 min. Note that Sypro Orange fluorescence measurements of as-purified SOD1 proteins were very similar in MES buffer pH 6.3 and in 50 mM Tris-HCl, pH 7.5, 150 mM NaCl and 10% glycerol (data not shown). Well to well variation was assessed and s.d. was quantified to be less than 10% in the different wells of a 96 well plate (data not shown).

### Hydrophobic binding

Equal amounts of protein (10 μg) were incubated on 20 μl Phenyl Sepharose 6 Fast Flow (high sub, GE Healthcare) in 200 μl of the indicated buffer overnight at room temperature and washed in binding buffer. Total, bound and unbound (supernatant) proteins were resolved on NuPAGE 4-12% Bis-Tris gels (Invitrogen) and stained with Coomassie Brilliant Blue G-250.

### Electrophoresis

For native PAGE, samples were equilibrated in Laemmli loading buffer lacking SDS and reducing agents, analyzed on 12% Tris-HCl gels and run in Laemmli buffer without SDS. NuPAGE 4-12% Bis-Tris gels (Invitrogen) were run in MES buffer and stained with Coomassie Brilliant Blue G-250.

### Aggregation analyses by DLS, ThT fluorescence, electron microscopy and filter retardation assays

Wild-type or mutant SOD1 (10 μM) were incubated at temperatures between 25 to 50° C, in the indicated buffers, in the presence of 0 to 20% of 2-2,2-trifluoroethanol where indicated (Fluka). After 4 to 5 hours (day 0) up to 7 days, the sizes of the particles were measured using the Zetasizer Nano S (Malvern) and presented either as the distribution of particle size or the average size (Z average). To monitor amyloid content, reactions were diluted into ThT solutions with a final concentration of 10 μM ThT and 30 mM glycine, pH 8.5. Fluorescence emission between 480 nm and 500 nm was measured immediately after dilution, using an excitation wavelength of 446 nm in a Tecan Safire II. Electron micrographs were acquired by using either a Philips EM208 or a Tecnai T12 transmission electron microscope. A sample of 2.5 μl of a 10 μM protein solution was negatively stained with equal volume of a saturated solution of uranyl acetate. Filter retardation assays were performed as described in^52^ and revealed with SOD-100E antibody (Stressgen).

Note that Sypro Orange is not present during any aggregation reactions.

## Appendix A

Supplementary Information

Supplementary Fig. 1. Characterization of purified SOD1. (a) NuPAGE analysis of 1.2 μg of purified SOD1^WT^ and its mutants. Note that some mutants migrate differently from SOD1^WT^, in agreement with previous studies.^1^ (b) Circular dichroism analyses of SOD1 proteins showing the characteristic spectra with the typical shoulder at 230 nm for the metallated SOD1 proteins.^2^ Solid lines: WTL and WT. Dashed lines: MBR mutants. Supplementary Fig. 2. Increased surface hydrophobicity upon thermal denaturation of ALS-causing SOD1 mutants. (a) Sypro Orange fluorescence during thermal unfolding of as-purified SOD1 and (b) EDTA-treated SOD1. Data are means and s.d. of 3 independent experiments. Supplementary Fig. 3. Metal contents of as-purified SOD1 variants and comparison of metal contents, hydrophobicity and apparent Tm, derived from Sypro Orange analysis. (a) Copper (Cu) and Zinc (Zn) contents assessed by ICP-MS expressed as metal equivalents per dimer, considering that fully metallated SOD1 would contain 2 Cu and 2 Zn equivalents per dimer. (b) Hydrophobicity of mutant SOD1 relative to wild-type (Δhydrophobicity), measured by thermal unfolding in the presence of Sypro Orange (Fig. 2) and metal content. (c) apparent Tm values of SOD1 variants obtained from thermal denaturation in the presence of Sypro Orange. (d) Hydrophobicity of mutant SOD1 relative to wild-type (Δhydrophobicity) and melting temperature (ΔTm), measured by thermal unfolding in the presence of Sypro Orange, between SOD1 mutants and SOD1^WT^. Supplementary Fig. 4. As-purified SOD1^WT^ and SOD1^A4V^ do not aggregate at pH 6.3. Analyses of SOD1 particle size by DLS. Supplementary Fig. 5. Aggregation of SOD1 mutants monitored by DLS (a, c) and Congo Red binding (b, d). SOD1^A4V^ were aggregated at pH 3.9 for 3 days or at 50°C for 5 days. A shift in the absorbance maxima suggests the presence of amyloid structures. Supplementary Fig. 6. Aggregation of as-purified ALS-causing SOD1 mutants is caused by their increased propensity to expose hydrophobic surfaces. (a) Correlation between surface hydrophobicity measured by Sypro Orange fluorescence and aggregation measured by DLS. Aggregation is expressed as the percentage of particles with a diameter larger than 12.5 nm. (b) Congo red binding to aggregated SOD1 proteins. Proteins were exposed to TFE for 1 day. A shift in the absorbance maxima suggests the presence of amyloid structures.
