# Supplementary material for: Exposure of Hydrophobic Surfaces Initiates Aggregation of Diverse ALS-Causing Superoxide Dismutase-1 Mutants
Source: J Mol Biol. 2010 Jun 11;399(3-3):512–25. doi: 10.1016/j.jmb.2010.04.019 (PMC2927901; doi:10.1016/j.jmb.2010.04.019)
Supplement: Supplementary Information [file mmc1.doc]

**Supporting Information**

**Circular dichroism**

Spectra (190–260 nm) of 18 μg of SOD1 proteins in 5 mM Tris-HCl, pH 8.0, 50mM NaCl were recorded on a JASCO J-810 spectropolarimeter.

**Metal content**

The metal content of as-purified SOD1 proteins was assessed by inductively coupled plasma mass spectrometry (ICP-MS) by Dr Jason Day, Department of Earth Sciences, University of Cambridge.

**Congo Red binding**

Aliquots of aggregation reactions were taken and mixed with 10 μM Congo Red. Absorbance spectra were measured in a Tecan Safire II.

1. Hayward, L. J., Rodriguez, J. A., Kim, J. W., Tiwari, A., Goto, J. J., Cabelli, D. E., Valentine, J. S. & Brown, R. H. J. (2002). Decreased metallation and activity in subsets of mutant superoxide dismutases associated with familial amyotrophic lateral sclerosis. *J Biol Chem* **277**, 15923-15931.

2. Arnesano, F., Banci, L., Bertini, I., Martinelli, M., Furukawa, Y. & O'Halloran, T. V. (2004). The unusually stable quaternary structure of human Cu,Zn-superoxide dismutase 1 is controlled by both metal occupancy and disulfide status. *J Biol Chem* **279**, 47998-48003.
