# Supplementary figures and images for: Exposure of Hydrophobic Surfaces Initiates Aggregation of Diverse ALS-Causing Superoxide Dismutase-1 Mutants
Source: J Mol Biol. 2010 Jun 11;399(3-3):512–25. doi: 10.1016/j.jmb.2010.04.019 (PMC2927901; doi:10.1016/j.jmb.2010.04.019)

(a)

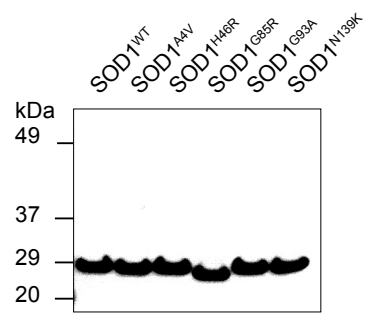

(b)

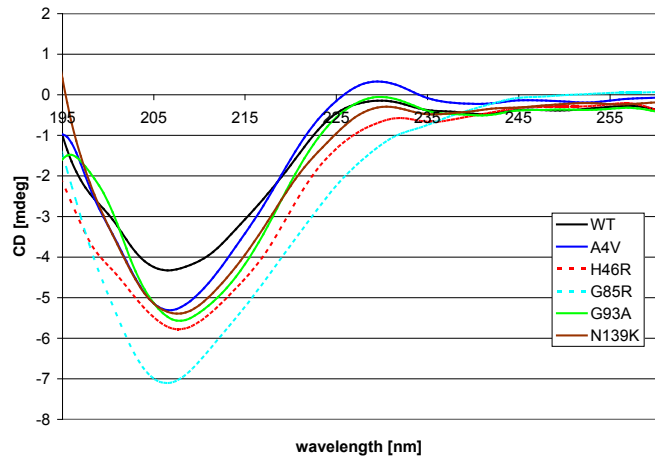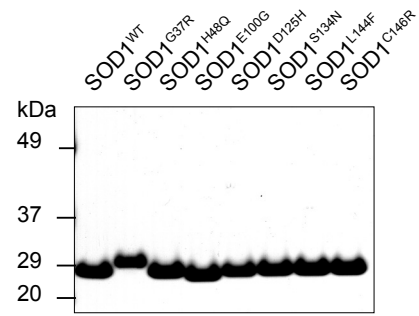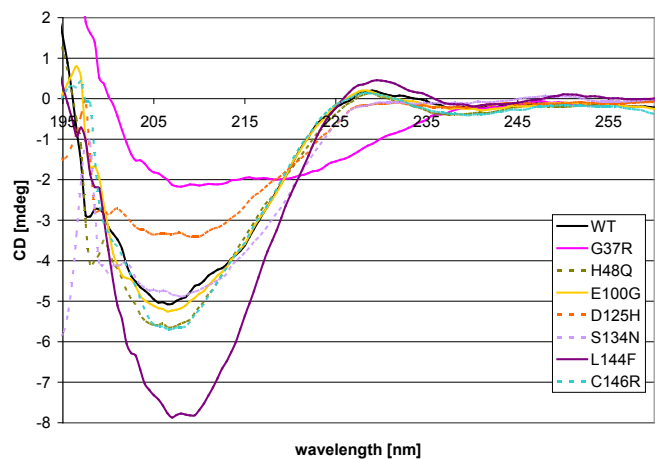

(a)

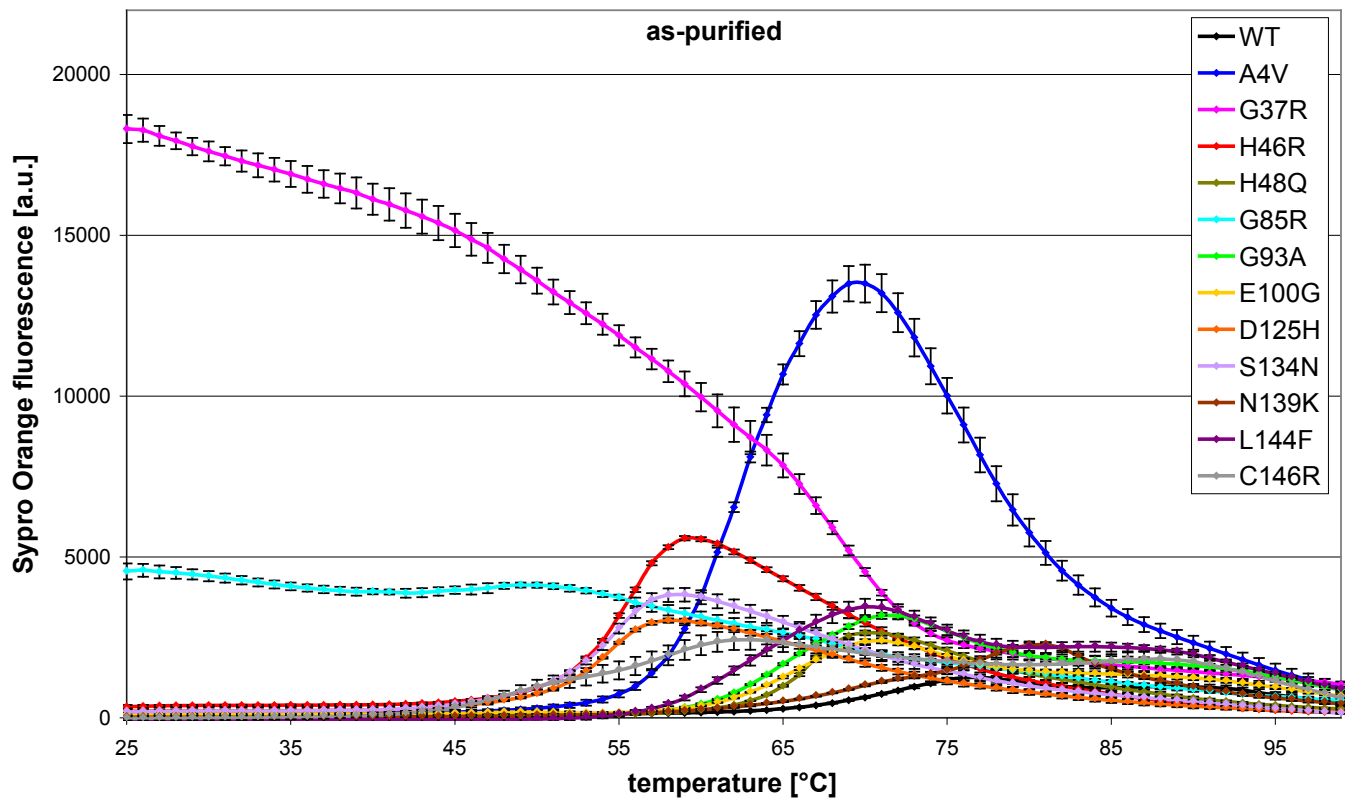

(b)

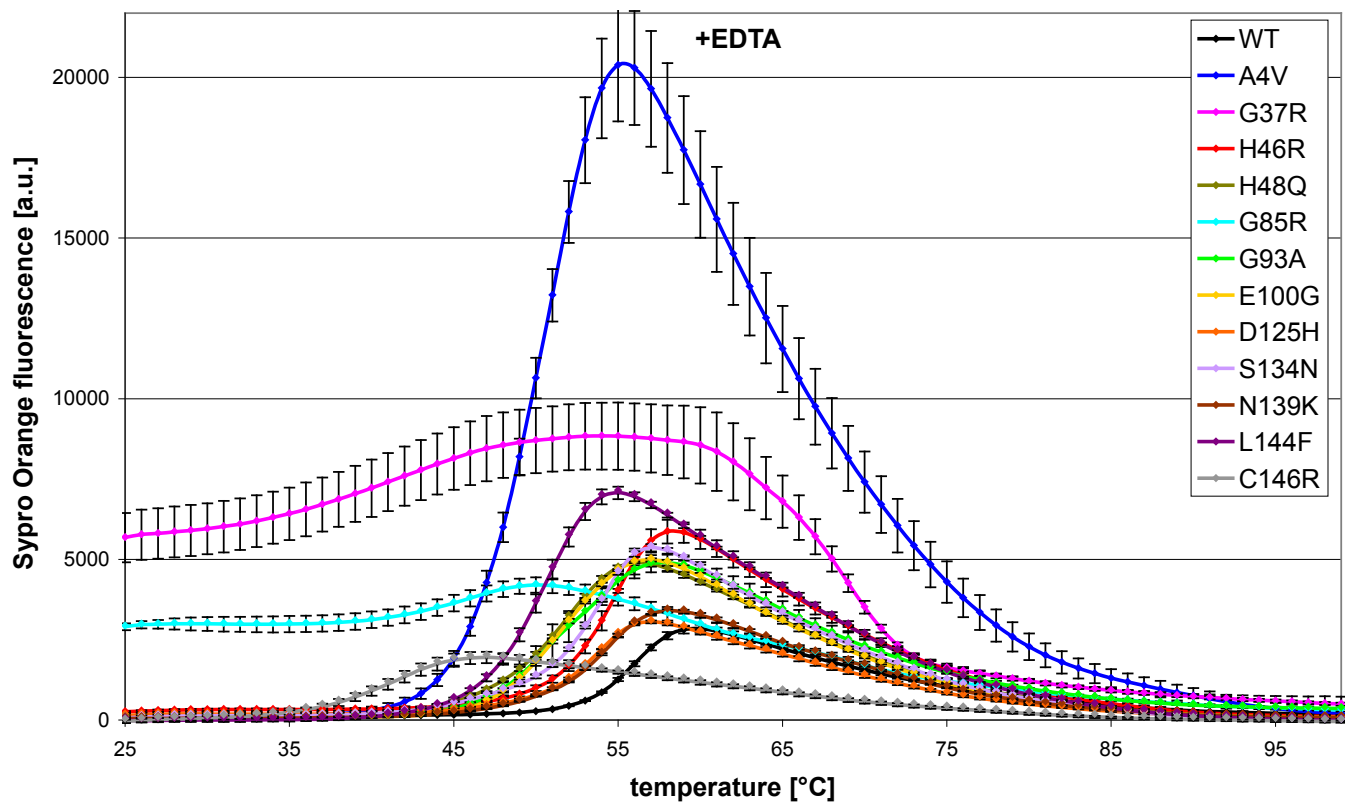

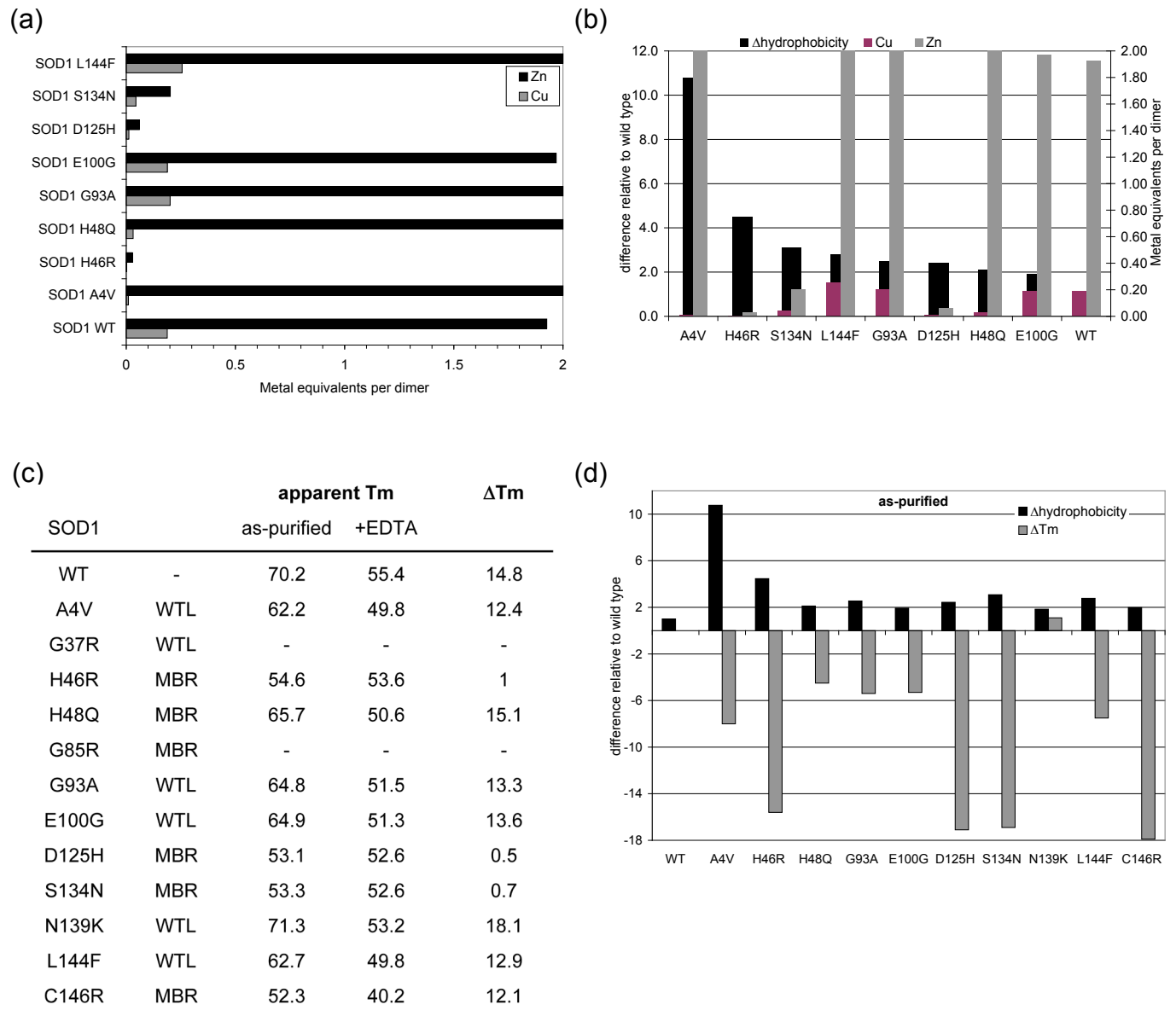

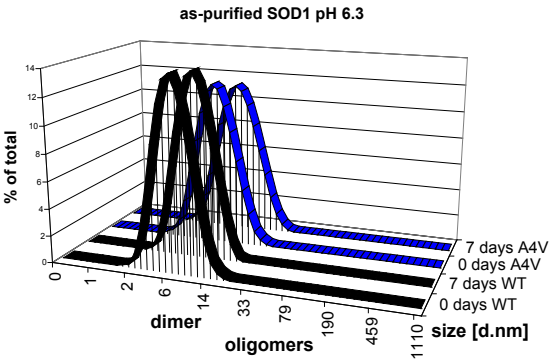

(a)

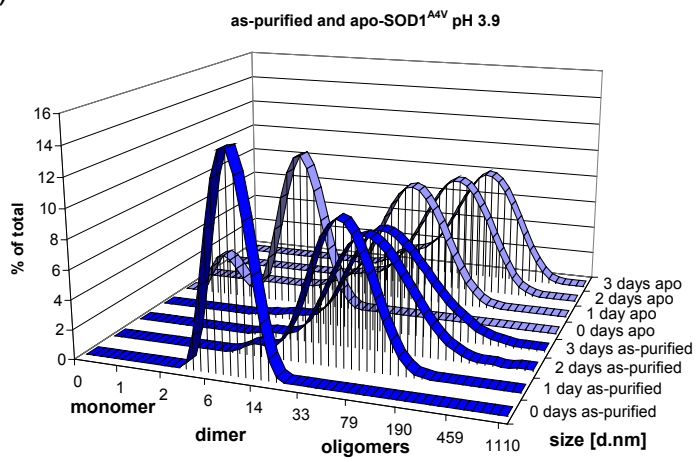

(b)

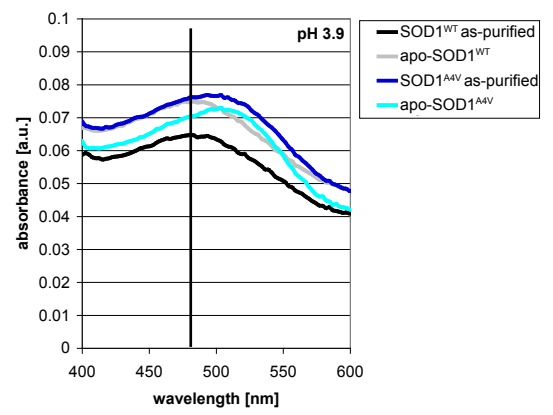

(c)

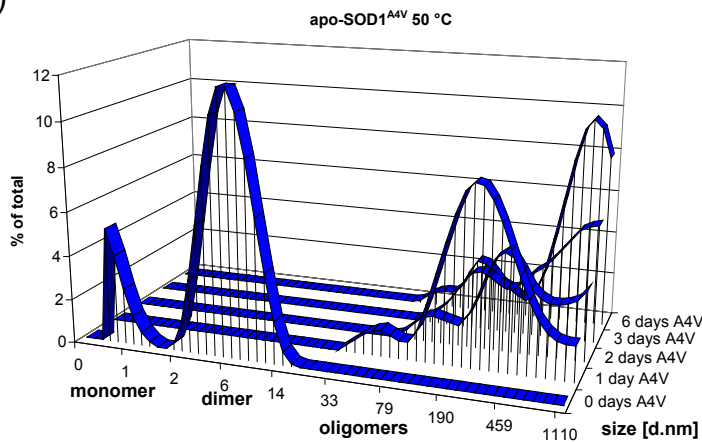

(d)

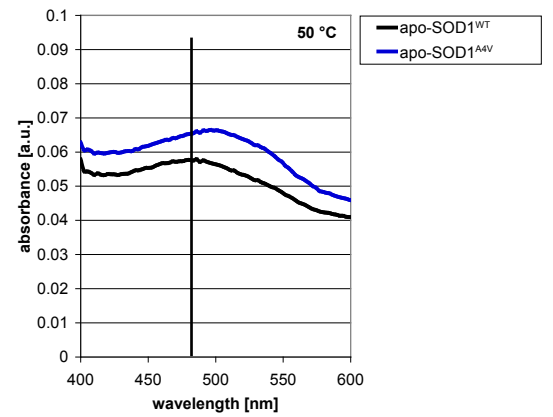

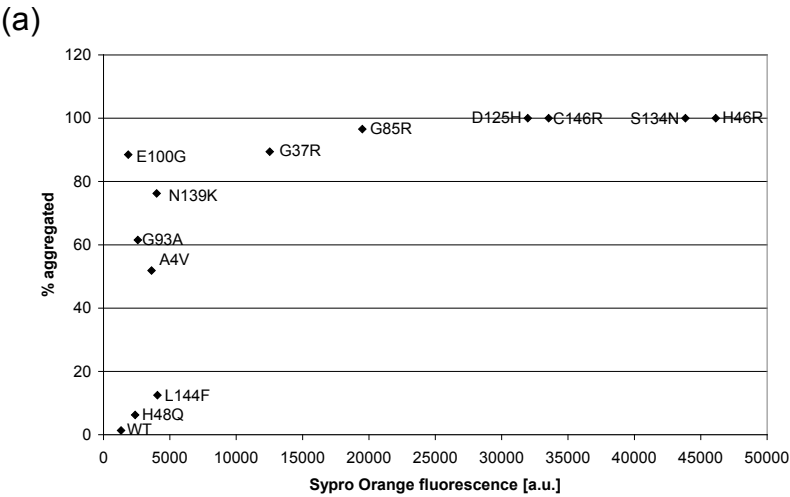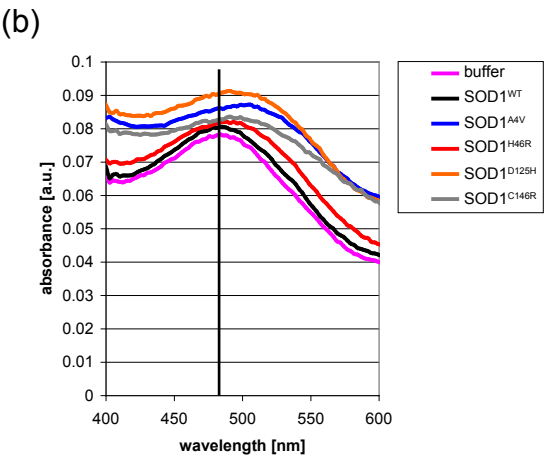

Supplement: Supplementary file 2 — Supplementary Fig. 1. Characterization of purified SOD1. (a) NuPAGE analysis of 1.2 μg of purified SOD1WT and its mutants. Note that some mutants migrate differently from SOD1WT, in agreement with previous studies.1 (b) Circular dichroism analyses of SOD1 proteins showing the characteristic spectra with the typical shoulder at 230 nm for the metallated SOD1 proteins.2 Solid lines: WTL and WT. Dashed lines: MBR mutants. Supplementary Fig. 2. Increased surface hydrophobicity upon thermal denaturation of ALS-causing SOD1 mutants. (a) Sypro Orange fluorescence during thermal unfolding of as-purified SOD1 and (b) EDTA-treated SOD1. Data are means and s.d. of 3 independent experiments. Supplementary Fig. 3. Metal contents of as-purified SOD1 variants and comparison of metal contents, hydrophobicity and apparent Tm, derived from Sypro Orange analysis. (a) Copper (Cu) and Zinc (Zn) contents assessed by ICP-MS expressed as metal equivalents per dimer, considering that fully metallated SOD1 would contain 2 Cu and 2 Zn equivalents per dimer. (b) Hydrophobicity of mutant SOD1 relative to wild-type (Δhydrophobicity), measured by thermal unfolding in the presence of Sypro Orange (Fig. 2) and metal content. (c) apparent Tm values of SOD1 variants obtained from thermal denaturation in the presence of Sypro Orange. (d) Hydrophobicity of mutant SOD1 relative to wild-type (Δhydrophobicity) and melting temperature (ΔTm), measured by thermal unfolding in the presence of Sypro Orange, between SOD1 mutants and SOD1WT. Supplementary Fig. 4. As-purified SOD1WT and SOD1A4V do not aggregate at pH 6.3. Analyses of SOD1 particle size by DLS. Supplementary Fig. 5. Aggregation of SOD1 mutants monitored by DLS (a, c) and Congo Red binding (b, d). SOD1A4V were aggregated at pH 3.9 for 3 days or at 50°C for 5 days. A shift in the absorbance maxima suggests the presence of amyloid structures. Supplementary Fig. 6. Aggregation of as-purified ALS-causing SOD1 mutants is caused by their increased propen [file mmc2.pdf]
